# The spectrum of pneumonia among intubated neonates in the neonatal intensive care unit

**DOI:** 10.1038/s41372-024-01973-9

**Published:** 2024-05-02

**Authors:** Dayle J. Bondarev, Rita M. Ryan, Devashis Mukherjee

**Affiliations:** Division of Neonatology, Department of Pediatrics, Rainbow Babies and Children’s Hospital, Case Western Reserve University School of Medicine, Cleveland, OH USA

**Keywords:** Respiratory tract diseases, Bacterial infection

## Abstract

We review the pathophysiology, epidemiology, diagnosis, treatment, and prevention of ventilator-associated pneumonia (VAP) in neonates. VAP has been studied primarily in adult ICU patients, although there has been more focus on pediatric and neonatal VAP (neo-VAP) in the last decade. The definition as well as diagnosis of VAP in neonates remains a challenge to date. The neonatal intensivist needs to be familiar with the current diagnostic tools and prevention strategies available to treat and reduce VAP to reduce neonatal morbidity and the emergence of antibiotic resistance. This review also highlights preventive strategies and old and emerging treatments available.

## Introduction

Invasive mechanical ventilation (IMV) through an endotracheal tube (ETT) continues to progress and evolve in neonatology. It is still a challenge to avoid ventilator-induced lung injuries (VILI) such as volutrauma, barotrauma, and atelectotrauma, inducing lung remodeling and repair. Despite advances in non-invasive ventilation (NIV), IMV is unavoidable for some neonates. Prolonged IMV can lead to VILI, pulmonary interstitial emphysema (PIE), air leaks, subglottic stenosis, as well as ventilator-associated pneumonia (VAP) [1].

The definition of VAP in the neonatal world remains unclear. The Centers for Disease Control and Prevention (CDC) have defined VAP as pneumonia associated with the use of IMV for at least two consecutive days [2]. The CDC and the National Nosocomial Infections Surveillance (NNIS) criteria both define VAP in infants less than one year old who require invasive mechanical ventilation (IMV) for 48 h, but they do not have definite criteria for newborns or premature neonates. The CDC’s definition for VAP states that IMV for at least 2 consecutive days is required for this diagnosis, along with demonstration of worsening gas exchange and evidence of the radiographic finding of new or worsening pulmonary infiltrate [2]. The current CDC criteria for VAP defined for an infant ≤1 year old must include worsening gas exchange and at least 3 of the following: temperature instability, leukopenia or leukocytosis, cough, new onset purulent sputum, change in character in sputum or increase in respiratory secretions, apnea, increased work of breathing, wheezing, rales or rhonchi and bradycardia or tachycardia (Table 1). VAP is a clinical entity that neonatologists must manage and still needs a proper national definition.

**Table 1:** CDC 2015 criteria for Diagnosing Ventilator- Associated Pneumonia (VAP) in <1 year old infants.

Definition	Signs/Symptoms	Imaging
Ventilator-associated pneumonia (VAP): Diagnosis of pneumonia in a patient on mechanical ventilation for >2 consecutive calendar days on the date of event, with the day of ventilator placed on the day of or the day before diagnosis of VAP	Worsening gas exchange (O_2_ desaturations, increased FiO_2_ requirement, or increased ventilator demand) ***AND*** at least three of the following: • Temperature instability • Leukopenia (≤4000 WBC/mm^3^) or leukocytosis (≥15,000 WBC/mm^3^) and left shift (≥10% band forms) • New onset of purulent sputum or change in the character of sputum, increased respiratory secretions, or increased suctioning requirements • Apnea or signs of respiratory distress • Wheezing, rales, or rhonchi • Cough • Bradycardia (<100 beats/min) or tachycardia (>170 beats/min)	Two or more serial chest imaging test results with at least one of the following: New and persistent **or** Progressive and persistent • Infiltrate • Consolidation • Cavitation • Pneumatoceles, in infants ≤1 year old
**Adapted for NICU patients (Neo-VAP)**^**a**^		
Same as above	Increased respiratory support + new chest X-ray findings + 3/5 of the following • Temperature instability • Tachy- or bradycardia • Clinical respiratory distress • Increased ETT secretions • Abnormal WBC	

Adapted from the Centers for Disease Control and Prevention (CDC) [2].

*WBC* white blood cell, *NICU* neonatal intensive care unit, *ETT* endotracheal tube.

^a^see Fig. 3*,* adapted from Goerens et al. [5].

The incidence of VAP in the neonatal ICU is dependent on the gestational age (GA) of the population, the presence of high-risk patients (such as patients with bronchopulmonary dysplasia, sedation requirements or a history of multiple intubations) as well as geographic location [3, 4]. In addition, reporting the incidence can be a challenge as the definition of neo-VAP can vary within different institutions. In developed countries, the reported incidence of VAP is between 5.8 and 19.7 episodes per 1000 ventilator days, compared with 37.2 per 1000 ventilator days in developing countries [5]. The variability in this incidence may be attributed to the lack of a clear definition of neonatal VAP (neo-VAP). In addition, although neonatologists at various institutions often use the CDC criteria for infants <1 year of age for the diagnosis of neonatal VAP, some institutions use their own criteria. This may contribute to the difference in incidence among centers.

This review aims to present the scientific literature on neo-VAP, including its pathogenesis, diagnosis, outcomes, preventive strategies, and old and emerging treatments available.

## Pathogenesis

**Fig. 1:**
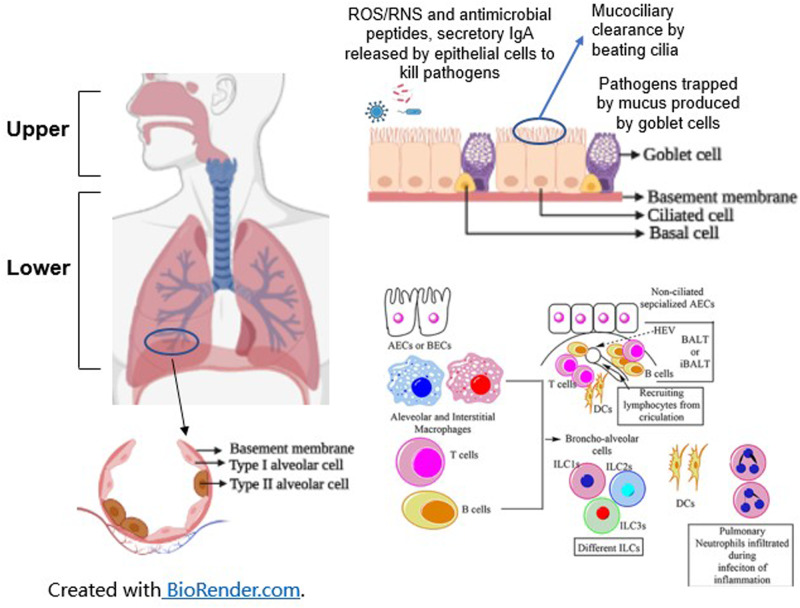
Mechanisms of airway pathogen clearance in upper and lower respiratory tract. ROS reactive oxygen species, RNS reactive nitrogen species, AECs alveolar epithelial cells, BECs bronchial epithelial cells, ILCs innate lymphoid cells, DCs dendritic cells, HEV high endothelial venule, BALT bronchus-associated lymphoid tissue, iBALT BAL induced in response to infection. Adapted from Kumar V, Front. Immunol 2020 and Adivitiya et al. Biology (Basel) 2021 [86, 87]. Certain parts of this image have been created with BioRender.com.

Pathogenesis of VAP in neonates is multi-factorial. Figure 1 shows the key innate and adaptive immune mechanisms that prevent upper and lower respiratory tract infections. Neonates are predisposed to nosocomial infections due to their immature immune system [6]. In addition, the skin and mucous membranes of premature neonates are not as effective in preventing infection as full-term neonates due to their increased permeability [7]. Premature neonates have an inadequate reserve of preformed neutrophils to mount an immune response, have immature granulocyte migration and bacterial phagocytosis, and have decreased immunoglobulins, as most transfer of immunoglobulins occurs during the third trimester of pregnancy [8, 9]. Lastly, there is evidence to show that the airway epithelium in preterm neonates is both functionally and structurally altered after preterm birth. There is goblet cell hyperplasia, leading to mucus hypersecretion and further airway obstruction, thickening of the ciliated portion of the epithelium, and increased number of apoptotic cells. Airway obstruction secondary to hypersecretion can decrease airway clearance and increase predisposition to VAP [10].

Endotracheal intubation impairs the natural defense system of mucus clearance. It connects the lungs with the oropharynx, eliminating the glottis’s natural barrier and allowing easy transfer of oral pathogens into the lower respiratory tract [11]. Duration of IMV is an independent risk factor associated with increased risk of VAP in neonates [12]. The oropharynx and pulmonary airways are colonized by non-pathogenic bacteria, which rarely cause disease. Antibiotic use and critical illness result in an abnormal host environment, leading to loss of normal flora and overgrowth of pathogenic species [13, 14]. These factors combine to predispose premature intubated neonates to acquire lower respiratory tract infections (Table 2).

**Table 2:** Risk Factors for Neo-VAP related to prematurity.

Non-modifiable	Modifiable
• Premature airway epithelium	• Ventilator days
• Immature immune system	• Prolonged antibiotic use
• Increased skin permeability	• Aspiration of gastric contents

*Staphylococcus* and *Ureaplasma* are some of the earliest colonizers of the upper airway tracts, appearing within the first five days of life in preterm mechanically ventilated infants [15]. Other bacterial pathogens which colonize neonatal ETTs include *Klebsiella*, *Streptococcus*, and *Pseudomonas* [15]. A cohort of 71 neonates had tracheal aspirates obtained within seven days of age and grouped into different clusters of either pro-inflammatory state or anti-inflammatory state. The study revealed that while Staphylococcus was the predominant species, *Ureaplasma* was not detected in the anti-inflammatory samples, suggesting that *Ureaplasma* may play a role in airway inflammation [16]. Collectively, these findings suggest that *Staphylococcus* is a common colonizer that serves as a commensal organism, and there are potential organisms that elicit a pro-inflammatory state. The microbiology of the ETT tip in neonates correlates closely with the subglottic area rather than the oropharyngeal area [17]. In summary, no particular organism is predominant in neo-VAP; hence, antimicrobial therapy reflects coverage for most gram-positive and gram-negative organisms.

As the upper respiratory airway tracts are constantly exposed to pathogens (either naturally or from nosocomial sources such as suctioning, infant handling, etc.), innate and adaptive immune defenses work in synchrony to clear the respiratory tract of microbes to enable adequate air exchange (Fig. 1). In preterm neonates, lung injury from prolonged ventilation and physical blockage of the endotracheal tube can inhibit pathogen clearance in the lower airway tract, allowing the proliferation of microbes, inflammation, and infection.

Another possible etiology for VAP is that reflux from the gastric fluid can cause gastric bacteria to transit into the airways [18]. This theory remains controversial as studies in the adult and pediatric populations have not consistently proven that acid-suppression prophylaxis prevents VAP [19–22]. The concept that microorganisms from the gastrointestinal tract can be present in the trachea is corroborated by data presented previously from El Abiad et al. in a single-center study in which there was 71% concordance between the organisms isolated from tracheal and gastric aspirates [22, 23].

Biofilms are structured communities of bacteria enclosed in a polymeric matrix adherent to an inert or living surface [13]. Any medical device or implant is a source of biofilm formation and thus can be a permanent source of infection. Intubation bypasses the upper airway, which decreases upper airway bacterial clearance and allows direct passage to the lower respiratory tract, which helps the ETT to act as a reservoir for biofilm formation [24, 25]. Suctioning the ETT can detach the biofilm from the ETT, which can then migrate towards the lower respiratory tract by the ventilator’s positive pressure. Feldman et al. showed that the secretions and airway access tubing lining the interior distal third of the ETT formed a biofilm [26]. The microflora in the ETT biofilms have shown an abundance of *Streptococci* in the neonatal ETT biofilms and is significantly related to the onset of VAP and colonization by other nosocomial pathogens such as *Pseudomonas aeruginosa* [27].

## Diagnosis

**Fig. 2:**
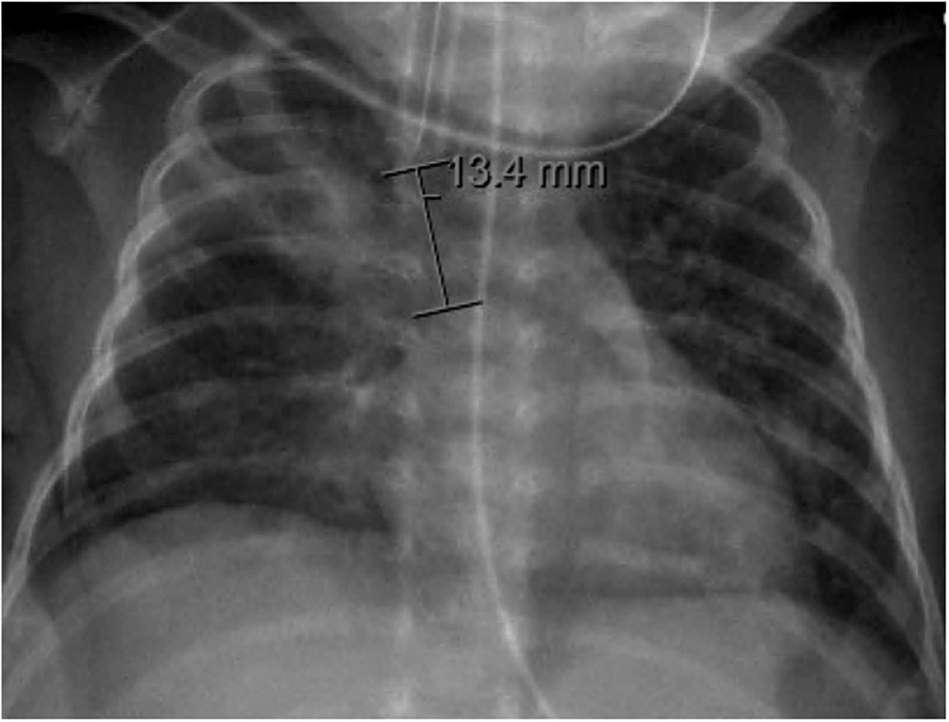
Chest radiograph images of neonates diagnosed with ventilator-associated pneumonia based on CDC guidelines. This radiograph was used to diagnose a ventilator-dependent 5-week-old, 27-week GA male with *Klebsiella pneumoniae* and *Staphylococcus aureus* pneumonia. CDC Centers for Disease Control and Prevention, GA gestational age.

There is no consensus statement from the American Academy of Pediatrics Section on Neonatal Perinatal Medicine or the Committee on Infectious Diseases on the definition of VAP in neonates. The CDC’s definition of VAP includes both clinical and radiographic findings. It is important to note that the rapid resolution of radiographic findings suggests a non-infectious process, such as atelectasis or congestive heart failure, and is inconsistent with VAP [28]. Frequent chest X-rays can be helpful since a longer course of antibiotics could be avoided when rapid resolution of lung “infiltrates” occurs. Chest radiograph findings can range from focal consolidates to subtle areas of infiltrates (Fig. 2).

Prematurity, longer hospital length of stay, and low birth weight are among the risk factors for VAP identified in the preterm population [12]. Sedation has also been associated with an increased risk of VAP, perhaps due to less ability to take deep sighs or to clear the airways [3]. Although multiple reintubation attempts have been associated with increased VAP incidence, this is likely due to a positive association between a higher number of reintubation attempts, increased IMV days, and lower birth weight, both of which are independently associated with increased VAP incidence [29].

Per CDC guidelines, at least one of the following must be collected to identify the causative organism for VAP: blood culture, pleural fluid, quantitative culture from minimally contaminated lower respiratory tract specimen such as broncho-alveolar lavage (BAL), protected specimen brushing or endotracheal aspirate [2]. In adults, BAL and bronchoscopic brushings are commonly used to collect airway samples to avoid contamination from the upper respiratory tract [30]. However, these techniques must be performed with bronchoscopic video guidance, which can be challenging to perform via the smaller endotracheal tubes and airways of neonates, and, often, appropriately sized equipment is a limiting factor. While tracheal aspirates (TAs) are more easily obtained from neonates, results can yield non-infectious colonizers, leading to unnecessary antibiotic usage, the development of antibiotic resistance, and the emergence of multi-drug resistant (MDR) organisms [31]. Also, evidence shows that TAs growing bacteria do not always represent clinical infection. In a retrospective cohort study in a level IV neonatal intensive care unit (NICU) where TA cultures were obtained in intubated neonates, positive cultures were not significantly associated with clinical, laboratory, or radiographic markers used to screen for infection [32]. Non-bronchoscopic BAL or “deep pulmonary lavage,” which employs blind wedging of the catheter below the carina, is also an option for obtaining samples that is a relatively safe diagnostic tool [33–35]. Although the utility of this technique in neonates is unclear, it has been used in randomized trials [36, 37]. Regardless of the sampling method, distinguishing VAP from colonization can be challenging. A cohort of preterm infants with bronchopulmonary dysplasia (BPD) requiring IMV for 21 days with serial TAs obtained at 72 h, seven days, 14 days, and 21 days of age showed that early bacterial colonization with diverse species is present within the first three days of life [38].

The CDC defines purulent sputum as secretions that contain ≥25 neutrophils and ≤10 squamous epithelial cells per low-power field. If the laboratory cannot provide additional information on quantitative reporting, they suggest direct examination results of “many,” “heavy,” “numerous 4+,” or ≥25 neutrophils per low power field to be interpreted as purulent secretions [26]. Alriyami et al. recommend the collection of TAs before and 24 to 48 h after empiric antibiotic treatment to assess changing microbiological patterns, including a decrease in colony counts or whether a polymicrobial sample shifts to a dominant organism, which is suggestive of resistance [22].

Obtaining a blood culture in the setting of pneumonia is controversial. The multicenter Etiology of Pneumonia in Community (EPIC) study showed that bacteremia was uncommonly detected in pediatric patients with community-acquired pneumonia and concluded that blood culture was low yield but could have utility in patients with parapneumonic effusion and intensive care unit (ICU) admission [39]. No study to date assesses the utility of blood culture in the setting of presumed VAP in preterm neonates. However, due to increased predisposition to infection in preterm neonates and the fact that the presentation of sepsis is similar to VAP in neonates, most neonatologists obtain blood cultures when there is a concern for VAP. Obtaining a blood culture will also allow a shorter antibiotic course if the culture does not detect bacteremia.

In a prospective study of extremely preterm neonates who were diagnosed with VAP using CDC guidelines, the most common pathogenic micro-organisms identified from respiratory secretions were *Pseudomonas aeruginosa, Enterobacter spp*., and *Klebsiella* for Gram-negative bacteria, and *Staphylococcus aureus* and *Enterococcus spp*. for Gram-positive bacteria [40]. In another study of neonates with a definite diagnosis of VAP (defined using the CDC criteria), 25% were polymicrobial VAP. Clinical features, therapeutic responses, and outcomes did not differ between monomicrobial and polymicrobial VAP in this cohort. Therapeutic response was defined by lack of treatment failure. Treatment failure of VAP included neonates who died from VAP, required antibiotics for more than two weeks, progression to bacteremia, and clinical deterioration after seven days of effective antibiotic treatment [41].

Endotracheal fungal colonization can also be found in 8.3–42% of intubated premature neonates [42]. Anti-fungal coverage should be considered for neonates not responding to antibacterial drugs or if the neonate has hyperglycemia, thrombocytopenia, and signs of skin involvement [43].

Specific biomarkers for pneumonia have been extensively studied in adults and may be helpful in neonates. C-reactive protein (CRP) is a well-studied marker in neonates and is used as a biomarker in sepsis [44]. CRP is a pentraxin protein produced by hepatocytes as an indirect response to inflammation. As tissue damage occurs, cytokine release triggers the hepatocytes to produce CRP that peaks within 4-6 h upon the onset of inflammation [44]. Single-center studies have shown that serum CRP is elevated in the majority of neonates diagnosed with VAP [45, 46]. The BioVAP study is a prospective multicenter observational study that evaluated the utility of CRP and procalcitonin in adults who received mechanical ventilation for >72 h. Their results showed that CRP and its rate of change per day (CRP ratio) were the best predictors of VAP in their patient cohort [47]. In a prospective cross-sectional study among 320 neonates with a cut-off value of 3.6 ng/mL, pooled sensitivity was 78%. At the same time, specificity was 70% in diagnosing neonatal sepsis [48]. Brown et al. performed a meta-analysis of 2 225 infants with sepsis and reported a median specificity of CRP of 74% with a median sensitivity of 62%, concluding that CRP may not be a valuable tool to withhold antibiotics considering an ongoing infection [49]. Both studies reflect that CRP can potentially be used in the correct clinical context of sepsis. Hence, the absence of CRP elevation does not rule out sepsis if the clinical picture suggests otherwise. Perhaps the trend of CRP can be used to guide treatment efficacy. In addition, it is essential to note that these studies are focused on neonatal sepsis as opposed to VAP. No studies have investigated the sensitivity and specificity of CRP in neo-VAP.

Another biomarker that could be utilized to diagnose VAP is procalcitonin (PCT). PCT was initially found to be increased in patients with staphylococcal toxic shock syndrome, and this resulted in numerous studies to assess the utility of PCT as a biomarker for sepsis [50]. PCT is a prohormone secreted as part of the inflammatory response to endotoxins in response to bacterial infection. In bacterial infection, inflammatory cytokines (such as tumor necrosis factor-alpha, IL-1β, and IL-6) induce gene expression responsible for PCT production. Cytokines that are selectively increased in response to viral infection do not cause the same upregulation of PCT compared to specific bacterial cytokine markers [51]. PCT can be used reliably to rule out bacterial infection [52]. In a multicenter randomized controlled trial (NeoPIns), 1 408 neonates were randomized either to PCT-guided decision-based versus standard care-based antibiotic treatment for early-onset sepsis, and the results showed that neonates enrolled under PCT-guided decision-making had a shorter duration of antibiotic use compared to the control group, without increase in any adverse events. Some neonatal and pediatric VAP studies have shown that PCT has an 80–90% sensitivity [53, 54]. A meta-analysis of 39 studies comparing PCT and CRP revealed that the mean sensitivity of early-onset sepsis (EOS), late-onset sepsis (LOS), and both EOS and LOS combined are 73.6%, 88.9%, and 76.5% respectively suggesting that procalcitonin is superior to CRP. However, only four studies included VLBW in their analysis [55]. This poses the same dilemma as CRP as these studies reflect neonatal sepsis, as opposed to neo-VAP. However, PCT may have a role in monitoring ongoing infections.

Presepsin is another potential biomarker/indicator of early-onset sepsis and has been evaluated in the diagnosis of VAP in the adult and pediatric populations. Presepsin is a product of the cleavage of CD14 from bacterial proteases during sepsis [56]. A recent meta-analysis of presepsin utility in diagnosing early-onset neonatal sepsis showed high sensitivity (93%) and specificity (91%), and its accuracy was not affected by the GA of the patients [57]. A cross-sectional observational study performed in intubated neonates from whom TAs were analyzed for presepsin showed that TA presepsin suggested the presence of early-onset pneumonia based on CDC criteria [58]. These studies show some promising utility of presepsin and its association with neo-VAP.

Point-of-care ultrasound is increasingly used for the diagnosis of VAP as an interpretation of radiographs can be challenging in patients with underlying chronic lung disease. When lung ultrasound was studied as a potential diagnostic tool for VAP, it showed a sensitivity of 94% with an area under the curve of 0.97. In this study, neo-VAP was determined by a multi-parameter ventilator-associated pneumonia score adapted from Goerens et al. and respiratory deterioration and confirmed by isolation of pathogenic microorganisms in airway aspirate. The criteria for lung ultrasound findings concerning for neo-VAP include the presence of consolidation ( >0.5 cm), pleural effusion, arborescence, or linear dynamic bronchograms [59].

## Outcomes

VAP has been associated with increased healthcare costs. A study by Ratcheva et al. demonstrated that in a cohort of 107 neonates on MV for >48 h, the length of stay for patients who met the criteria for VAP was 32 days compared to 18 days for non-VAP patients. The median hospital cost for patients with VAP is €3675.77, compared to the lower expenses of €2327.78 for non-VAP patients (U = 1791.5, *p* < 0.001) [60]. In addition to increased healthcare costs, VAP is also associated with higher morbidity and mortality. In a prospective cohort study in the US of 229 patients admitted to the NICU with a birth weight ≤ 2000 grams receiving at least 48 h of IMV, 28.3% met the CDC VAP criteria for infants. The authors demonstrated a significant association between VAP and mortality in neonates who stayed in the NICU for greater than 30 days (RR 8 with 95% CI 1.9–35) [40]. This highlights the importance of monitoring VAP to decrease mortality. A prospective cohort study of 199 inborn neonates who were mechanically ventilated for 48 h was classified as either exposed or unexposed to VAP defined by CDC < 1-year-old criteria. The study revealed that the incidence of BPD was higher in VAP patients with an adjusted relative risk ratio of 3.5 (1.002–12.7, *P* = 0.049) and the number needed to harm of 2.07. However, the composite outcome of BPD/mortality did not differ. Infants diagnosed with VAP are also smaller in GA and have longer IMV duration, both of which are risk factors for BPD. This emphasizes the importance of hypervigilance with the diagnosis and treatment of neo-VAP in the ELBW population with pre-disposition to BPD. In addition, VAP-exposed neonates had a longer length of stay (87 [43–116] vs 14 [8–52] days, *P* < 0.0001) as well as fewer ventilator-free days (22 [14–24] vs 11 [5–17.7] days, *P* = 0.05) compared to non-exposed neonates, which remained statistically significant after adjusting for GA. Respiratory infections, rehospitalization, and home oxygen therapy were similar in VAP and non-VAP cohorts [61]. In another prospective study by Wang et al., neonates with polymicrobial VAP were shown to have a significantly increased incidence of neurological sequelae with an adjusted odds ratio of 2.74 (95% CI 1.18–6.36, *P* = 0.019) [41].

The emergence of MDR organisms can be a sequela of utilizing broad-spectrum antibiotics to treat VAP in the NICU. Wang et al. reported the incidence of MDR neo-VAP was 39.2%, and these neonates were shown to have a longer duration of antibiotic use and delayed resolution of symptoms [62]. This highlights the importance of identifying microbes involved in VAP to narrow antibiotic coverage to prevent the emergence of MDR organisms. This is one example in which TA cultures can be helpful.

## Management

Understanding the microbiology of VAP is essential to making an informed decision on empiric antibiotic treatment, followed by narrowing it down to specific targeted therapy based on microbial sensitivities or discontinuing antibiotics.

Most management data in neo-VAP is focused on neonatal sepsis rather than VAP. In addition, there are no uniform recommendations regarding antibiotic guidance for neo-VAP, and most centers use empiric antibiotic therapy in VAP as they would for early or late-onset neonatal sepsis. Current recommendations are to use ampicillin and gentamicin for early-onset sepsis (within 72 h of birth) and nafcillin and gentamicin for late-onset sepsis [63]. Broad coverage for the source of infection targeting known typical nosocomial flora, antibiotic susceptibilities of previous infections, and antibiogram patterns for each NICU should all be considered. Empiric antibiotics for late-onset infection should cover *Staphylococcus* species and Gram-negative bacteria such as *Pseudomonas aeruginosa, Klebsiella spp, Escherichia coli*, and *Serratia marcescens*. If there is an associated risk of aspiration, anaerobic coverage should also be considered. In infants who do not seem to be improving, a third or fourth-generation cephalosporin (e.g., ceftazidime or cefepime) with pseudomonal coverage is recommended, as well as adding vancomycin for methicillin-resistant staphylococcal coverage. When these higher-level antibiotics are being used, it is prudent to involve pediatric infectious disease specialists with expertise in antimicrobial stewardship to limit antibiotic resistance in the NICU.

Inhaled antibiotics reduce systemic toxicity as they are given locally with limited systemic absorption. Nakwan et al. reported treatment of *Acinetobacter baumanii* in a small cohort of preterm neonates using aerosolized colistin for 72 h as an adjunct to standard intravenous antibiotic treatment [64]. This was a retrospective matched case-control study in 16 neonates with MDR Gram-negative VAP and showed that neonates who received both intravenous and inhaled colistin had a higher clinical cure and microbial eradication, along with lower ventilator requirements at the end of treatment as compared with neonates receiving intravenous colistin alone [65]. These studies show some clinical evidence for adding an inhaled antibiotic, but a more extensive cohort study is needed to confirm clinical efficacy. However, it is crucial to recognize that inhaled or oral drugs are not typical treatments for neonatal pneumonia in the setting of presumed sepsis. Although it is used in some NICUs, there has not been a large cohort study on inhaled antibiotics in preterm neonates to assess their efficacy and toxicity.

**Fig. 3:**
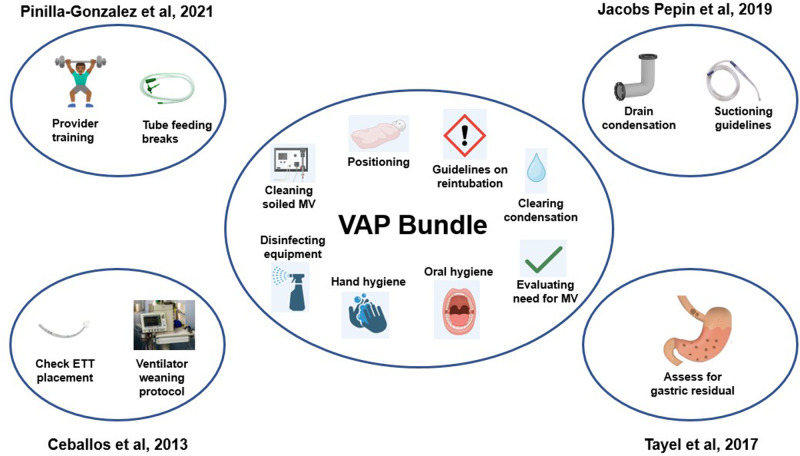
Common quality improvement measures and bundles used in neonatal intensive care units to prevent VAP. The four studies on the periphery depict the most commonly cited studies on the effectiveness of VAP bundles. In the center, we list the measures widely used by NICUs as a part of VAP bundles [73–76].

There is no consensus on the duration of treatment of neo-VAP. However, Goerens et al. provided a recommendation for suspected VAP and length of treatment based on clinical status and laboratory findings of (1) worsening clinical and ventilation conditions, (2) abnormal laboratory findings (CRP > 20 mg/l), leukopenia (≤4000 WBC/mm3) or leukocytosis (>15,000 WBC/mm3) and left shift (>10% band forms), (3) and positive cultures from tracheal secretions. If all three criteria are met, the authors recommend a duration greater than or equal to 7–14 days of antibiotic therapy. A treatment of 24–36 h plus an additional 48–72 h would be warranted if the patient only shows 1–2 of the criteria, and discontinuation of antibiotics after 24–36 h if only 0–1 of the criterion is met [5]. Figure 3 shows a modified adaptation from Goerens et al. which can serve as a clinical decision-making tool for the neonatologist at the bedside. It is important to note that this is a proposed algorithm, and data has not been published to assess its efficacy. The clinical rounding team should frequently discuss the duration and choice of antibiotic therapy with the clinical pharmacist. In addition, consulting the pediatric infectious disease team and referring to local antibiogram patterns of each NICU is also warranted. It is worth mentioning that emerging studies show that shorter durations of antibiotics might be as effective as longer durations and can reduce changes in the emergence of antimicrobial resistance [66, 67]. Lewald et al. performed prospective surveillance of infants diagnosed with pneumonia and sterile blood culture receiving a five-day course of antibiotics where 14% of their cohort were infants ≤37 weeks. Their study revealed that only 3% of their cohort had antibiotics restarted after five days for relapsed pneumonia. Three infants died within 14 days of antibiotic discontinuation from Pseudomonas sepsis, and one infant died from *Klebsiella* sepsis due to necrotizing enterocolitis NEC [68]. The significance of this study is that it shows the potential for shorter course treatment. However, further studies in premature neonates need to be conducted.

## Prevention

Prevention of VAP is accomplished by avoiding prolonged IMV. Removing the ETT when possible is critical in preventing VAP and should be a focus of everyday clinical decision-making. The CDC and Healthcare Infection Control Practices Advisory Committee (HICPAC) recommends using orotracheal tubes and changing respiratory circuits only for malfunction or contamination. There is no recommendation for routine exchange of ETTs or circuits without any clinical indication [69].

Hand hygiene is an important strategy to prevent nosocomial infections such as VAP. In a study in which alcohol-based hand gels were used for hand hygiene, a reduction of neo-VAP was shown in VLBW neonates [70]. In another study of NICU patients, increased compliance with hand hygiene reduced the incidence of respiratory infections from 3.35 to 1.06 per 1000 patient days [71]. Suctioning of the endotracheal tube is done to clear airway secretions. No specific recommendations on types or frequency of airway suctioning have been shown to decrease the incidence of neo-VAP for neonates. Cordero et al. have shown no difference between open and closed suctioning in neonates regarding VAP incidence or mortality, even though it seems logical that a closed system should be helpful [72].

**Fig. 4:**
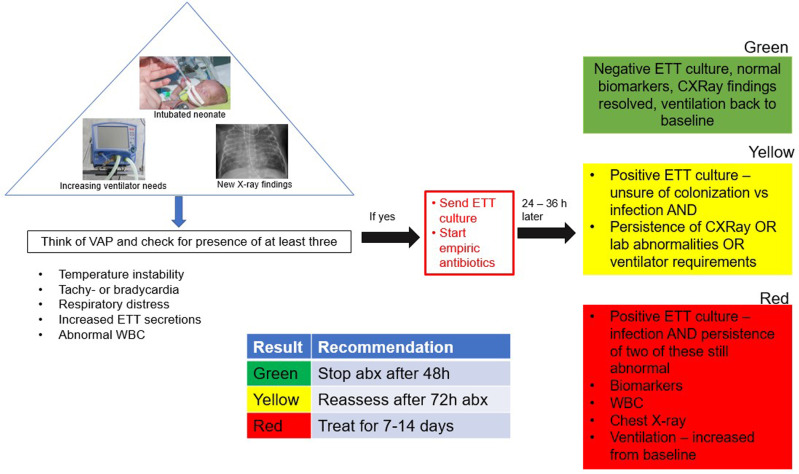
Bedside algorithm for the diagnosis and treatment of neonatal VAP. Flowsheet for the neonatal provider at the bedside to aid in diagnosing and managing VAP in the neonate based on CDC guidelines and published studies and modified from Goerens et al. Front Pediatr 2018 [5].

Studies on VAP in neonates focusing on prevention are limited to quality improvement projects and generally focus on implementing multiple-item bundles (Fig. 4). Azab et al. performed a prospective observational cohort study of 143 neonates, implementing a bundle that included frequent assessment for extubation readiness, hand hygiene, oral hygiene, positioning, closed sterile suctioning, strict reintubation guidelines, and changing the ventilator circuit every seven days. Their bundle showed a significantly decreased incidence of neo-VAP from 36.4 to 23 cases per 1000 IMV days (*P* = 0.0006) [73]. Jacobs Pepin et al. enforced a similar NICU bundle with the addition of guidelines on disinfecting the patient environment and draining condensation from ventilator tubing when suctioning and showed a decrease in the incidence of neo-VAP from 8.5 to 2.5 cases per 1000 ventilator days (*P* < 0.004) [74]. Ceballos et al. demonstrated that in addition to the common interventions for the prevention of hospital-acquired infection in the NICU, replacing the circuit with reintubation, ensuring a sterile field upon intubation, frequent endotracheal tube positioning, a protocol for weaning off the ventilator decreased their incidence of neo-VAP from 8.9 to 3.9 cases per 1000 vent days in neonates <750 g [75]. Lastly, a prospective cohort study by Pinilla-Gonzales et al. introducing a similar bundle with the addition of sterile airway management and feeds running for 60–120 min as opposed to bolus or continuous feeds showed a decrease of VAP incidence from 11.7 to 1.9 cases per 1000 ventilator days (*P* < 0.01) [76].

Elevating the head of the bed is a part of some neo-VAP prevention bundles. This originated from adult literature proposing that VAP is associated with supine patient positioning, which promotes gastroesophageal reflux with subsequent aspiration. Gastric content refluxing into the airway is believed to increase the risk of VAP, as gastrointestinal pathogens can colonize the airway. However, head of bed elevation is against safe sleep recommendations by the American Academy of Pediatrics. Bagucka et al. showed that head-of-bed elevation can promote reflux in term infants compared with supine positioning [77, 78]. In the pediatric literature, there is no significant difference in VAP incidence between patients who receive H2-blocker prophylaxis compared to patients who did not [79]. In addition, the use of histamine H2-receptor antagonists (H2-blockers, e.g., ranitidine) in neonates is controversial since recent studies have shown that the use of H2-blockers could be associated with NEC, sepsis, osteoporosis, and intraventricular hemorrhage [80].

Several other VAP preventive measures include using bacteriostatic nanotechnology-coated ETTs and probiotics. In adults, bacteriostatic nanotechnology coating can be used on the ETT to prevent biofilm formation. However, this has not been studied in the neonatal population, and efforts are underway to adapt this technology potentially [81]. Sun et al. performed a systematic review and meta-analysis in adults, children, and neonates on the efficacy and safety of probiotics in preventing VAP. It showed evidence to suggest that prophylactic probiotics could help prevent VAP [82]. In one large Level IV NICU, placement of ultra-violet germicidal irradiation into the heating-ventilation-air conditioning circuit significantly decreased the VAP rate [83]. There is also evidence that oropharyngeal colostrum could significantly reduce VAP and NEC [84]. Lastly, there is emerging evidence for using nebulized hypertonic saline for airway clearance. A small cohort study in premature infants showed that nebulized hypertonic saline decreases VAP occurrence [85].

## Conclusion

Neo-VAP is a challenging diagnosis given the current CDC definitions that are not specific to neonates, especially preterm neonates, who constitute most of the neo-VAP burden. The diagnosis and management of VAP have progressed minimally over the last decade despite medical and technological advancements in neonatology. There are few sensitive or specific markers for diagnosing pulmonary infection in neonates apart from culture, which has the challenge of separating invasive infection from colonization. Newer biomarkers such as procalcitonin and presepsin have been described but not proven superior to ETT culture. Differentiating colonization and infection presents a significant challenge for neonatologists in a baby with persistent radiographic infiltrates. It often leads to overuse of antibiotics, leading to the emergence of further antibiotic-resistant strains. There is a growing repertoire of VAP prevention bundles and emerging non-antibiotic prophylactic medications. However, there is still a need for better, earlier, and more definitive diagnoses, treatment, and prevention options for neo-VAP, given the associations with increased morbidity and mortality, as well as the contribution to increased length of stay and healthcare costs.
